# A Patient's Perspective on Health Information Management

**DOI:** 10.3928/24748307-20170307-03

**Published:** 2017-03-23

**Authors:** James H. Lytle

I have Parkinson's disease (PD), and the experience of learning to deal with it has made me aware of how complex managing health care information is. What has most surprised me since my diagnosis is the disconnect between providers who have responsibility for my well-being, my health information records, and the stance of my care team. In this essay, I use my experiences to explore what health literacy should mean for patients with a chronic illness, the problems with current health information systems, and how health care organizations need to design support systems with patients' interests foremost.

## My Patient Experience

In theory, I have access to one of the most sophisticated health care organizations in the United States—a research university health system that, like several of its peer institutions, uses a state-of-the-art electronic health record (EHR) as its information management system. I also have the additional advantage of being a relatively informed patient, as for much of my professional career I have managed large data systems, albeit in the K-12 public education sector.

As a patient of the university health system over the course of several decades, I use or have used the services of general medicine, internal medicine, cardiology, anesthesiology, neurology, dermatology, surgery, orthopedics, gastroenterology, otolaryngology, allergy, dentistry, and physical therapy. I have online access to a portion of my medical records. A reasonable assumption would be that the array of those who provide my health care, and particularly my primary care physician, would all have access to information related to the range of services and medications I am using or have used. I am aware of privacy protections afforded by HIPAA (Health Insurance Portability and Accountability Act), but would assume that my informed consent should make all of the information regarding my health readily available to all of my providers. But, as I have learned, that is not how it works in practice, as a few examples from my own experience make clear.

Although my dentist is affiliated with the university and can prescribe whatever medications I might require, she has no access to my complete health records but only the information that I have chosen to share with her. Conversely, none of my health system providers have access to my dental records.

Another example relates to physical therapy. As someone with PD, I benefit from regular exercise and have developed a daily routine with the help of a therapist located at the neurology practice. However, she is employed by an independent organization that operates on a contract with the university, and so has no access to my health records, even though she is the one who is most likely to note changes in my physical abilities, such as balance, flexibility, strength, or coordination. That information, however, is only made available to my neurologist if I act as the transmitter.

Then there is the matter of my urologist and ophthalmologist, who are not a part of the university system and don't have access to the university's EHR, so each depends on me for pertinent health information. Any diagnoses, procedures, or prescriptions they make only enter my base file if I make the request to my primary care physician.

A fourth example is more arcane. When I recently needed a hernia operation, I was required to have a cardiac assessment because of an abnormality with my electrocardiogram test. The resident who interviewed me acknowledged that various departments in the health system, including hers, took a proprietary stance on their patient data, and that she had to sign off one record system and then sign into another to complete my preoperative assessment.

## My Records—What's There and What's Missing

When I access my university medical record system account, I am given the option of managing appointments or renewing prescriptions, and reviewing my current health issues, medications, health summary, and medical history. When reviewing my account recently, I was surprised to learn that PD is not listed as part of my medical history, although it is included in my health summary. The date of my initial Parkinson's diagnosis is included, but when I access that line I am sent to a proprietary site that provides generic information but no details regarding my specific case. The medication list includes rasagiline (with the trade name Azilect [Teva Neuroscience, Overland Park, KS]) as a subtitle; two clicks/screens later I can learn about the side effects and interactions of Azilect, but the record system does not make that process obvious or simple.

My medical history record shows “broken collarbone” and my surgical history shows “orthopaedic surgery,” but there is no indication that the two are connected, that I had two surgeries on the collarbone because the first didn't work, and that I now have a metal plate holding the collarbone together. Nor does my record indicate anything about my heart stress test. (I might add that I've never been offered a tutorial on how the site works, nor is one available on the site.)

What I have come to realize from these experiences is how dysfunctional my health records system is, and how important it is to maintain my own health records, particularly because I have a progressive disease. It is the aggregate of my health information that allows me to monitor and determine how I am doing. As is evident in a PD support group I attend, many of my fellow PD patients trust that their health systems and providers are keeping track of them by coordinating the health information available from their many and various providers, when that is not necessarily the case.

## My Concerns

As I think about it, I'm struck that the health care record system, at least the one I have access to, is designed as an accounting, marketing, liability protection, departmental control, and communications system, but not as a comprehensive and responsive information system, and that there is an asymmetrical relationship between me and my care team. They have access to more elaborate versions of my health records than I do, and that raises a seminal question: whose information is it? It's my body and my health, so shouldn't the system be designed so that I have full access, so I that can be as fully informed as possible, and so my university-based and nonuniversity-based providers (and emergency department doctors) can have immediate access to the entirety of my records? Is the university's system designed to ensure its ownership and control of my records, thereby increasing my dependency on their health system and my continuing patronage? (In the current health system parlance, I am a “customer,” a term that makes my dermatologist cringe while acknowledging that he's just had to attend a workshop on “customer relations.”)

## Curing vs. Engaging

In trying to make sense of my health systems experiences, I turned to a book by Ron Heifetz (1994), a psychiatrist who teaches leadership at Harvard University's Kennedy School of Government. In *Leadership Without Easy Answers* he describes three types of problems that leaders, doctors, and patients face:

- Type I – situations in which the patient's expectations are realistic: the doctor can provide a solution and the problem can be defined, treated and cured, assuming the patient's compliance.
- Type II – “the problem is definable, but no clear-cut solution is available.” Although the doctor may play a central role, the patient has to recognize and own his problem sufficiently that he makes the life changes his improved health requires. The doctor needs to help the patient do the work that only the patient can do.
- Type III – “situations are more difficult. The problem definition is not clear-cut, and technical fixes are not available. The situation calls for leadership [by the doctor] that induces learning even when the doctor does not have a solution in mind. Chronic illnesses fit this category. The doctor can continue to operate in a technical mode by diagnosing and prescribing remedies, yet doing so avoids the problem-defining and problem-solving work of both doctor and patient. The patient's real work, [hopefully with the doctor's assistance] is facing and making adjustments to the harsh realities that go beyond his health condition.” (Heifetz, 1994, pp. 74–76)

As a chronic illness, PD is clearly a Type III challenge—I'm not going to get better and there is no cure. My neurologist monitors my symptoms and progression, but I am not being taught how to approach the “harsh realities” I face.

## My Learning Resources

Interestingly, my PD patient support group, organized by the neurology practice at which I am a patient but managed by the participants, is often my best information source. But none of the neurology doctors attend as observers, even though it could be a rich source for learning how their patients are dealing with PD (eg, marijuana use).

To date, I have reason to believe that my PD symptoms are evolving slowly, and after 3.5 years I'm still taking a low-impact medication with no apparent side effects. But how long will that pattern hold? As I hope I've made clear, I have a sense of the range of my health information, but how much of that does my neurologist need to know? And who among my health providers is going to take the time to teach me what I need to keep track of, and how to manage my life when my PD is confounded with aging? Do they see themselves as tutors, teachers, and collaborators in the manner that Heifetz's Types II and III problems suggest? Or is it that they don't have time, interest, or an orientation toward teaching rather than unilateral problem solving?

## My Questions

Is it my responsibility to insist that my doctors, and in particular my neurologist and primary care physician, take time to review the entirety of my records and educate me? Should it be the role of my primary care physician to act as an information gatekeeper and manage my complete health care, ensuring that there are no conflicts in the medications I'm getting and that everyone who needs to is attending to my health care data? Does HIPAA provide a convenient (but unwarranted) excuse for my health care providers to avoid obtaining the information they might need to safely and effectively provide treatment? Why is this whole thing so fragmented, and does anyone even care?

I've looked at Internet-based personal health record maintenance forms that might help me, but the ones I've reviewed are not as comprehensive as they need to be and have inherent biases. Both my spouse and I are still able to monitor our health conditions and advocate for ourselves, and as a manifestation of our relative privilege, we have long-term care insurance that includes the services of a health care advocate with access to the medical records we provide her. Is this sufficient? I don't know, but I'd be more comfortable knowing that all my health records were readily available to my entire care team, including those unaffiliated with the university.

## My Perspective

Over time I've found that my support group, the social worker who attends the meetings, and the publications and websites of the Parkinson's Foundation (http://www.parkinson.org) and the Michael J. Fox Foundation (https://www.michaeljfox.org) are my best learning resources. I now recognize how little of what the group members share is learned from their neurologists and primary care doctors, and how much they depend on the Internet and social media discussion groups for information. I also continue to wonder how patients who have less access to health data and information than I do manage their health.

That raises the big question: What does “health literacy” mean for me and others with a chronic illness? I should think it means what literacy means—competence or knowledge in a specific area. That suggests reorienting patients' expectations for a cure, and physicians' orientations from problem solvers to patient educators. It means simplifying and organizing medical records for ready access. It means putting in place monitoring systems that both patients and their care teams regularly access, a process made increasingly simple with wearable technologies. It means teaching patients what they need to know and do, and that requires an orientation on the part of the providers to health literacy, which in turn requires seeing health care from the patient's perspective.
